# The relationship between paediatric foot posture and body mass – do heavier kids really have flatter feet?

**DOI:** 10.1186/1757-1146-6-S1-O12

**Published:** 2013-05-31

**Authors:** Angela Evans

**Affiliations:** 1 Health, Rehabilitation and Research Institute, AUT University, Auckland, New Zealand; 2 Health Science, University of South Australia, Adelaide, 5000, Australia

## Background

The prevailing opinion is that heavier children have flatter feet, a consistent finding of the studies that have addressed this issue. Recently, we queried this observation and postulated that the method of foot posture assessment may influence this finding, as there was no positive relationship between the BMI and FPI-6 scores of 140 school children. Most other studies have used footprint-based measures.

## Methods

Data was acquired from four datasets from previous works, (South Australia, n=303; UK, n=225; rural South Australia, n=140; New Zealand, n=30) providing 698 observations of children’s BMI and FPI-6 scores. Descriptive statistics were used to examine the basic anthropometrical characteristics of the study populations. Parametric statistical correlations were applied to continuous data, and scatter plots were used to explore and illustrate relationships between parameters.

## Results

The total population results yielded the following mean (SD): age 9.20 years (2.34), BMI 18.29 kg/m^2^ (3.52), FPI-6 4.68 – 4.95 (3.12 – 3.31). Gender N = 698; 359M: 339F. Correlations between BMI and FPI-6 ranged from – 0.89 (p=0.05) to -0.115 (p=0.01) for the study population (n = 698) aged from 3 to 15 years. The mean population (n=698) BMI = 18.29 kg/m^2^ (3.51), whereas the mean BMI for the ‘flatfoot’ (FPI-6 ≥ 6, n=267) = 18.16 kg/m^2^ (3.73) and the ‘non flat-foot’ group BMI (FPI-6 < 6, n=431) = 18.51 (3.43).

**Figure 1:**
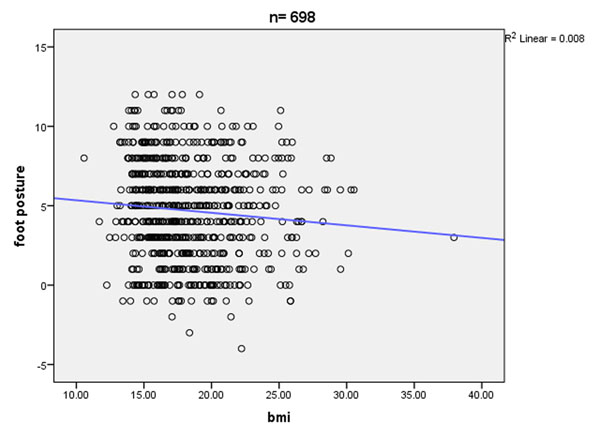
Foot posture versus body mass index

Using the international obesity task force cut-off points for overweight, the study population was also evaluated for BMI/foot posture (FPI-6) for each age year group.

## Conclusion

This study supports our earlier findings, and conflicts with many other studies, in not finding a positive correlation between increased BMI and ‘flatter’ feet in children. Clinically, these findings question the need for concern about children’s BMI as a specific influence on (flatter) foot posture, and also the validity of footprint based measures.

